# Central Dental Association of Northern New Jersey

**Published:** 1887-06

**Authors:** James G. Palmer


### CENTRAL DENTAL ASSOCIATION OF NORTHERN NEW JERSEY.

### Celebration of its Seventh Anniversary.

BY JAMES G. PALMER, D. D. S.

Early in the year 1880 a few gentlemen practicing dentistry in and near Newark, N. J., met to consider the advisability of forming a local society. Some years before such an organization had been organized. It was, however, "born but to die." The meeting at which it came into existence, and a subsequent one, when it was wound up, are its only records. With this as a precedent, the gentlemen persevered, and on the 19th of May, 1880, the "Central" was duly incorporated by gentlemen from Newark, Elizabeth, Orange, Jersey City and New Brunswick.

At the time of its organization, the State Dental Society was in a flourishing condition. The location of the State, between New York City on the one hand and Philadelphia on the other, the advantages derived from association with professional brethren of these cities, and the feeling of pique at the frequent taunt of being "from Jersey," from whence no good was likely to come, combined to produce in the minds of the incorporators of the "C. D. A." a determination to succeed—to show other societies that New Jersey could do as well as any. They labored hard, and the society grew apace. Seven years have passed, and to-day we embrace in our membership the leading members of our profession throughout the northern and middle portions of the State. So interwoven with the interests of the State Society have become the interests of the "C. D. A.," that it almost goes without saying that to be a member of one is to be a member of both. Indeed, all the members of the "C. D. A." are members of the State Society.

Thursday evening, May 19, 1887, was celebrated for the first time, in a public manner, the anniversary of the incorporation of our society. At 8.30 P. M. there gathered around the table, spread in the dining-room of the Park House, in Newark, a goodly number of the members of the C. D. A. and invited guests. Among the latter were Drs. Northrop, Kingsley, Rich, Weld, Walker, Day, Gage, Atkinson, Nash, Parr and others of New York; Bonwill, of Philadelphia; Knapp, of New Orleans, and many more.

After the feast physical, came the intellectual. President S. C. G. Watkins, as chairman and toastmaster, told a good story, welcomed the guests and announced the first toast, "Our Invited Guests," which was drunk standing.

"The New York State Dental Society" was then proposed, and Dr. N. W. Kingsley was called upon to respond. He commenced by saying: There *is* such an organization as the New York State Dental Society, and it is compossed principally of dentists. There are a few medical gentlemen who condescend to practice dentistry, and who are associated with us. We do not object; in fact, we like to have high-toned gentlemen with us. It inspires and encourages us, and makes us feel that we are in good society. At some future time, when we get through making money, we may choose to practice medicine ourselves.

The next toast announced was "The Odontological Society of Pennsylvania," and Dr. W. G. A. Bonwill was called upon and gave a brief sketch of that society. He regretted that it had not done as much in a social way as some of its younger sisters. It had not been its custom to invite men from other States to attend entertainments, but perhaps in time it would inaugurate something of that kind.

"The First District Dental Society of the State of New York" brought to his feet its President, Dr. W. W. Walker, who said that of all the societies with which he was connected that was the one with which he most delighted to work. He detailed some of the scientific work accomplished by its members, and said that he believed it had done as much as any other to place dentistry upon the high pinnacle on which it stands to-day.

"The Press" was fittingly represented by Mr. Howells, who read an appropriate poem.

The next toast was "The American dentist; the thinking man, the generous man. The greater the demands upon him the larger his generosity to his professional brethren." To this comprehensive toast Dr. Atkinson was called upon to respond. He was in a peculiarly happy humor and took a roseate view of affairs dental, as the following "Atkinsonian" from his speech will show:

"We need not go further to see that we are not a mere specialty in medicine, but the crowning excellence of all the modes of healing that have ever yet been pronounced among men, from the earliest solidal through the fluidal and the cellulal to the pragmatic pathology which was pronounced only on this planet after dentistry became a distinct calling, by reason of the neglect of naturalists, divines, lawyers, medical men and the common polity, and their ignoring the organs upon which we must spend our time, and in which all the principles of the organism are developed and portrayed, the interference with which modes of activity lays the foundation for the necessity for the calling known as dentistry."

"The New Jersey State Dental Society" was responded to by Dr. C. S. Stockton, who spoke of the fraternal feeling which was or should be engendered by society meetings, and paid a glowing tribute to the society which he was called upon to represent.

Dr. John B. Rich was called upon to speak of "Reminiscences of Dental Practice," and sketched the difference in the practice of fifty years ago and that of to-day.

"The Dinner Committee" was honored, and Dr. Levy in response said that he hoped that those who were invited and did not come, might never fully know what they had missed.

"The Central Dental Association" was the subject of the next toast, and Dr. C. A. Meeker spoke of the steps taken to organize and incorporate the society, which had grown to an importance which no one at that time anticipated.

At "Low Twelve" the company dispersed, having thoroughly enjoyed a very pleasant evening, and filled with the hope that the society might celebrate many such anniversaries.

